# Phenotyping of Macrophages After Radiolabeling and Safety of Intra-arterial Transplantation Assessed by SPECT/CT and MRI

**DOI:** 10.1177/09636897231212780

**Published:** 2023-11-27

**Authors:** Ida Friberger, Vamsi Gontu, Robert A. Harris, Thuy A. Tran, Johan Lundberg, Staffan Holmin

**Affiliations:** 1 Department of Clinical Neuroscience, Karolinska Institutet, Stockholm, Sweden; 2 Department of Neuroradiology, Karolinska University Hospital, Stockholm, Sweden; 3 Centre for Molecular Medicine, Karolinska University Hospital, Stockholm, Sweden; 4 Department of Oncology and Pathology, Karolinska Institutet, Stockholm, Sweden; 5 Department of Radiopharmacy, Karolinska University Hospital, Stockholm, Sweden

**Keywords:** endovascular, intra-arterial, MRI, SPECT/CT, rabbit

## Abstract

Cell therapy is an integral modality of regenerative medicine. Macrophages are known for their sensitivity to activation stimuli and capability to recruit other immune cells to the sites of injury and healing. In addition, the route of administration can impact engraftment and efficacy of cell therapy, and modern neuro-interventional techniques provide the possibility for selective intra-arterial (IA) delivery to the central nervous system (CNS) with very low risk. The effects of radiolabelling and catheter transport on differentially activated macrophages were evaluated. Furthermore, the safety of selective IA administration of these macrophages to the rabbit brain was assessed by single-photon emission computed tomography/computed tomography (SPECT/CT) and ultra-high-field (9.4 T) magnetic resonance imaging (MRI). Cells were successfully labeled with (^111^In)In-(oxinate)_3_ and passed through a microcatheter with preserved phenotype. No cells were retained in the healthy rabbit brain after IA administration, and no adverse events could be observed either 1 h (*n* = 6) or 24 h (*n* = 2) after cell administration. The procedure affected both lipopolysaccharide/gamma interferon (LPS/IFNγ) activated cells and interleukin 4 (IL4), interleukin 10 (IL10)/transforming growth factor beta 1 (TGFβ1) activated cells to some degree. The LPS/IFNγ activated cells had a significant increase in their phagocytotic function. Overall, the major impact on the cell phenotypes was due to the radiolabeling and not passage through the catheter. Unstimulated cells were substantially affected by both radiolabeling and catheter administration and are hence not suited for this procedure, while both activated macrophages retained their initial phenotypes. In conclusion, activated macrophages are suitable candidates for targeted IA administration without adverse effects on normal, healthy brain parenchyma.

## Introduction

In recent years, the field of regenerative medicine has expanded dramatically with the development of cell transplantations. Cell transplantations are used in a broad veracity of diseases with both stem cells and immune cells for the treatment of cancer, autoimmune diseases, and regeneration of damaged tissues^1–4^.

With the advent of more extensive clinical trials, translation from small rodents to humans has also brought new challenges to the field, such as scalability and covering a larger target area with the same cell graft density^5^. The development of cell therapy as an integral part of regenerative medicine has a long history^5^. During the past 15 years, a range of different cell types has been tested in several disease states, primarily in small rodents, with promising results including but not limited to stroke, diabetes mellitus, congestive heart failure, and cancer. A typical cell-based therapy is an injection of immune cells such as macrophages, T-cells, B-cells, and natural killer cells^6–11^. Macrophages are known for their sensitivity to activation stimuli and the capability to recruit other immune cells to the sites of injury and healing. These properties make macrophages viable treatment candidates in cancer and many other disease settings^12,13^. Clinical cell-based therapy has traditionally entailed the administration of monocytes, that is, naive macrophages^14,15^. These circulate in the blood until they reach a target tissue, where they may become differentially activated depending on the tissue stimuli, resulting in phenotypes that either drive or modulate the inflammatory process.

Compared with pharmacological treatment, in cell therapy, the route of administration requires more consideration due to the complexity of the cells’ biological characteristics, biodistribution, and tissue interactions^16,17^. The potential advantages of cell transplantation using an intra-arterial (IA) microcatheter are: (i) targeted delivery to the site of injury and consequently increased treatment efficacy and (ii) decreased off-target accumulation and therefore reduced risk of side effects. One disadvantage, compared with standard injections, is that it is significantly more invasive, hence the risk of tissue damage. Previous microcatheter-based cell delivery has proven to increase engraftment in organs such as the intestines and bone marrow^18^. Yet, the validation of microcatheter delivery of radiolabeled cells to the central nervous system (CNS) is currently lacking, such that the degree of *in vivo* cellular functionality post-transfer is unknown. The “cell quality” after radiolabeling has traditionally been assessed solely on the basis of viability staining, low-specificity biopsy staining, and treatment efficiency^19–21^. For the therapeutic efficiency of cell transplantation, it is thus crucial to ensure that the cells are unaltered by the delivery technique and maintain their functionality *in vivo*. Since a common cell type to administer is monocytes, it then implies that the cells reach their target site in a monocyte state. If cells instead were to be activated due to the administration technique, it could initiate an aggressive inflammatory response, hence damaging the tissue. This effect in some treatments is desirable while in others can cause severe complications and tissue damage or even support tumor growth^22–24^. If transplantation of unstimulated monocytes were to be used for cancer treatment, the desire would be for the cells to differentiate into lipopolysaccharide/gamma interferon (LPS/IFNγ) activated cells since they can cause an aggressive immune response against the tumor. If the monocytes were to differentiate into interleukin 4 (IL4), IL10/transforming growth factor beta 1 (TGFβ1) activated cells, it would trigger a regenerative response that would amplify the tumor growth.

The same applies to stem cell treatment, if the stem cells were to prematurely differentiate due to cell stress, losing their regenerative properties, it could jeopardize the treatment efficiency.

The proposition of tracking cells *in vivo* is not new, and several methods are currently available for this purpose. However, cell tracking using nuclear imaging techniques such as positron emission tomography (PET) and SPECT, combined with anatomical CT imaging, has the unique advantage of providing non-invasive, long-term biodistribution *in vivo* in real time^25^. For example, a common compound used for long-term nuclear cell tracking with SPECT imaging is the radiotracer (^111^In)In-(oxinate)_3_, an intracellular labeling agent with a half-life of 2.8 days^26–30^.

In this study, we aimed to evaluate the effects of the microcatheter cell delivery technique on human bone marrow-derived monocytes and monocyte-differentiated macrophages. The macrophage subtypes were either stimulated with LPS/IFNγ or IL4/IL10/TGFβ1 to differentiate into activated macrophages. To assess the effects of radiolabeling and delivery procedures on cells, we investigated the phagocytotic function and expression of phenotypic markers in both pre-radiolabeling and post-radiolabeling as well as pre-catheter and post-catheter passage. We further investigated the retention and possible adverse effects of IA administration of activated cells in healthy rabbit brains.

## Materials and Methods

### Cell Preparation

Human monocytes and macrophages were isolated from buffy coats donated by healthy volunteers (Clinical Immunology and Transfusion Medicine Department of Karolinska Institute, Sweden) (Ethical approval Dnr: 9328-2019), according to a previous publication^31^. Cells were purified from the peripheral blood layer of mononuclear cells (PBMC) using Ficoll-Hypaque separation (GE Healthcare, Chicago, IL) according to the manufacturer’s instructions. Monocytes were isolated from the PBMC using a CD14^+^ Selection Kit (Miltenyi Biotech, Bergisch Gladbach, Germany) and cultured in RPMI1640 medium with 10% FBS (Sigma-Aldrich, St. Louis, MO). The human monocytes were further differentiated into macrophages in the presence of M-CSF (50 ng/ml) (BD Pharmingen, San Diego, CA) and cultured for 6 days. Separate fractions of the human monocyte-differentiated macrophages were stimulated with either LPS/IFNγ or IL4/IL10/TGFβ1 (R&D Systems Inc, Minneapolis, MN) for 24 h as previously described^32^. All subtypes were analyzed with FACS for surface expression of immune markers.

### Cell

Radiolabeling of human macrophages with Indium (^111^In)In-(oxinate)_3_ was performed according to the product description of lymphocyte labeling (Mallinckrodt Medical, London, UK)^33^. Briefly, 37 MBq (^111^In)In-(oxinate)_3_ in Tris buffer was added to the cells (27–30 × 10^6^ cells, 1.3–1.2 Bq/cell) in a phosphate-buffered saline (PBS) (Sigma-Aldrich, St. Louis, MO) suspension. The cells were incubated for 20 min in a humidity chamber at 37°C, and the cells were gently mixed every 10 min. Subsequently, cells were washed twice with PBS (0.5 mL of 0.5 M PBS) at 1,000 rpm for 5 min and resuspended in warm PBS. Supernatants and cell pellets were measured for radioactivity in a dose calibrator (Capentec CRC-15R Dose Calibrator, ALT, New London, CT) to determine cell labeling efficiency. Cell viability was measured with trypan blue staining and analyzed using a Countess II Automated Cell Counter (Thermo Fisher, Boston, MA). Cellular retention has previously been evaluated^34–36^.

### An Endovascular Catheter Passage *In Vitro*

**Figure 1.**
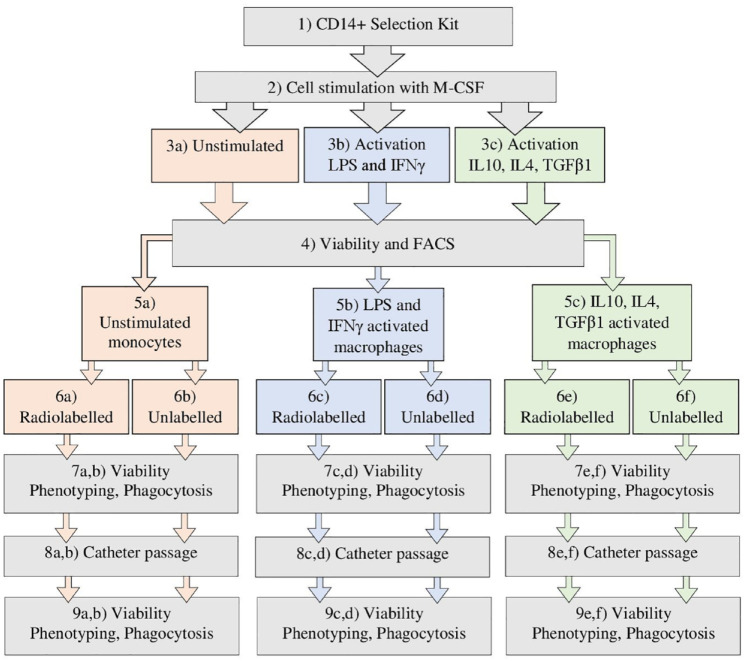
A step-by-step graphic chart over the *in vivo* validation process of the radiolabeling and catheter passage of unstimulated monocytes, LPS/IFNγ or IL4, IL10/TGFβ1 activated macrophages.

Triplets of all cell types (2.4 ± 1.1 × 10^6^ cells) suspended in 1 ml PBS were slowly passed through a 1.2 F microcatheter (Magic; Balt Extrusion, Montmorency, France), mimicking an approximately 4–8-minute *in vivo* injection, the catheter being washed with PBS after each cell passage. All unlabeled cell types were analyzed before and after passing through the microcatheter (Fig. 1). After the initial catheter passage, all cell types were then radiolabeled with (^111^In)In-(oxinate)_3_, and the procedure of catheter passage was repeated with analysis before and after passage through the catheter. This procedure provided data on all cell lines, both unlabeled pre-catheter and post-catheter passage and radiolabeled pre-catheter and post-catheter passage (Fig. 1). Cell samples were analyzed for (i) phenotype surface antigen expression, (ii) phagocytotic ability by flow cytometry, and (iii) viability by trypan blue staining.

### Phenotype and Phagocytosis by Flow Cytometry

Cell phenotypes were analyzed with an antibody panel design to differentiate between the cell types CD14-FITC, CD16-APC-H7, CD86-BV421, CD163-PE, and CD206-APC (BD, Franklin Lakes, NJ) (Table 1). The unstimulated M0 phenotype corresponds to a positive expression of CD14^++^, CD86^++^, and CD163^+^. The LPS/IFNγ activated cells phenotype shows a positive expression of CD14^++^, CD86^+^, CD163^+^, and CD206^+^. Finally, the IL4, IL10/TGFβ1 activated cells show a positive expression on CD14^++^ and CD206^++^. Phagocytosis was quantified by the uptake of Dextran Alexa Fluor™ 647 (Thermo Fisher, Boston, MA). Dextran and antibodies were added to samples of approximately 0.6–1 × 10^5^ labeled/unlabeled, pre-catheter/post-catheter cells in 100 µl PBS and subsequently incubated at either 37°C with Dextran or 4°C with antibodies for 30 min. Samples were analyzed using a flow cytometer (Guava HT12®, Merck, Rahway, NJ) and software (Guava HT12®, Merck easyCyt system). The cellular morphology of cell size and intracellular structure was evaluated by forward scatter (FSC) and sideway scatter (SSC).

**Table 1.** An Overview of the Antigens Used to Evaluate the Phenotypic Expression of Unstimulated Monocytes and Activated Macrophages and Their Role in Immune Cell Functions.

Antigen role in immune cell functions				
Antigen	Unstimulated	LPS/IFNγ	IL4/IL10/TGFβ	Target receptor purpose and response
CD14	++	++	++	Aka: lipopolysaccharide-binding protein. Detects bacterial antigens^37^.
CD16	–	–	–	Aka: FcγRIIIa receptor, antibody-dependent cell-mediated cytotoxicity. Positive for large granular lymphocytes T-cells and NK cells. The low expression on monocytes and macrophages^38^.
CD86	++	+	–	Aka: B7-2. Antigen-presenting cells for costimulatory signals necessary for T-cell activation. Positive on monocytes and LPS/IFNγ activated macrophages^39^.
CD163	+	+	–	Aka: hemoglobin scavenger receptor. The macrophage-specific protein responsible for inflammation type 1, binds TNF-α. Positive on monocytes and LPS/IFNγ activated macrophages^40,41^.
CD206	–	+	++	Aka: mannose receptor and C-type lectin, identify pathogens, active in type 2 immune response. Positive for IL4, IL10/TGFβ1 macrophages^42^.

Levels of expression are divided into high (++), low (+), and minimal expression (–). LPS/IFNγ: lipopolysaccharide/gamma interferon; IL4: interleukin 4; IL10: interleukin 10; TGFβ1: transforming growth factor beta 1.

### Animal Model

Eight female New Zealand White rabbits weighing 3.5 ± 0.3 kg were used in the study (Ethical approval Dnr: 5.2.18-15846/17) due to their large size and availability. Animals were housed 7–10 days prior to experimental day and fed food and water *ad libitum.* General anesthesia was induced by subcutaneous (SC) injection of 0.5 ml/kg Hypnorm (fentanyl citrate 0.315 mg/ml, fluanisone 10 mg/ml; Janssen Pharmaceuticals, Belgium) combined with 5 mg diazepam (Actavis Group PTC, Iceland). Rabbits were intubated and connected to a servo ventilator. Maintenance of anesthesia was achieved by continuous intravenous (IV) infusion of Propofol-Lipuro (Braun AB, Sweden) at a rate of 20 ml/h and IV injection of 0.1 ml Hypnorm every 30 min.

All large animal angiography and endovascular interventions were performed under sterile conditions using Philips XD20 angiographical equipment (Philips medical system, the Netherlands). Visipaque 270 contrast agent (GE Healthcare, USA) was used in all angiography applications.

A 4F pediatric short introducer (Terumo Medical Corporation, Japan) was inserted into the femoral artery through a surgical incision, and a 4F catheter (Vertebral; Cook, USA) was navigated to the common carotid artery. A 1.2F microcatheter (Magic; Balt Extrusion, France) was then navigated to the internal carotid artery, and in that position, 20 million cells were infused at a concentration of 10,000 cells per microliter with a total injection time of 8 min (*n* = 3 for LPS/IFNγ activated cells and *n* = 3 for IL4, IL10/TGFβ1 activated cells). Digital subtraction angiography was performed following the injection and was not repeated at later time points.

### Magnetic Resonance Imaging

Magnetic resonance imaging (MRI) was obtained within 15 min of cell administration for all animals, and repeated after 24 h for the rabbits that received [^111^In]In-(oxinate)_3_ labeled cells. The MRI was conducted at 9.4 T in a horizontal magnet with a 30-cm-wide opening (Agilent Inc, UK). A 20-cm gradient insert, capable of generating 30 G/cm gradient fields, was used together with a birdcage coil of 150 mm inner diameter for transmission (Rapid Biomedical, Germany).

In a supine position, the rabbits were placed in the MRI-compatible bed designed for obese rats (Rapid Biomedical, Germany). The head was secured in a semi-circular surface coil designed for rat heart imaging (Rapid Biomedical, Germany), which was used for signal reception. Diffusion-weighted imaging (DWI) was accomplished through a fat-suppressed spin-echo sequence [TR 2,900 ms, TE 22.96 ms, next 1, matrix size 128 × 96 field of view (FOV) 60 × 60 mm^2^, 27 contiguous slices of 1 mm thickness] with diffusion weighting gradients (18.64 G/cm, 23.8 ms, gradient separation 12.9 ms, *b*-value 951.8 s/mm^2^) applied along 12 directions and 2 reference images, where the diffusion weighting gradient intensities were set to 0. T1-weighted images were obtained using a fat-suppressed spin-echo sequence (TR 700 ms, TE 15.12 ms FOV 60 × 60 mm^2^, matrix size 192 × 192, 14 slices 1 mm with 1 mm gap, repeated in 2 min 17 s, repeated with the slice package translated 1 mm in the slice select direction to keep TR short to have T1 weighting and have complete coverage). Images with higher spatial resolution were acquired using a fast spin-echo 3D sequence (TR 800 ms, ETL 8, zero 4, effective TE 31.18 ms, FOV 51.2 × 51.2 × 51.2 mm^3^ matrix 256 × 192 × 192).

### SPECT/CT Imaging

Single-photon emission computed tomography/computed tomography (SPECT/CT) was obtained after 24 h post-injection of the two animals that received [^111^In]In-(oxinate)_3_ labeled cells. The SPECT/CT system (Symbia T; Siemens GmbH, Germany) consisted of a dual-head variable-angle γ-camera equipped with low-energy high-resolution collimators and a multislice spiral CT component optimized for rapid rotation. The SPECT acquisition (128 × 128 matrix, 81 frames, 45 s/frame) was performed using 4.5° angular steps in a 50-s time frame. For CT (158 kV, 210 mA, B50s kernel, 512 × 512 matrix), 0.75-mm slices were obtained. After reconstruction, SPECT images were corrected for attenuation and scatter. SPECT and CT axial 5-mm slices were generated using Hermes Gold 450 (Hermes Medical Solution, Sweden). Images were then analyzed with OsiriX imaging software (OsiriX Foundation, Geneva, Switzerland).

### Statistical Analysis

All data are presented as mean and standard deviation (SD) unless explicitly stated otherwise. Statistical significance for paired samples was calculated using the Student *t*-test and for repeated measurement, the repeated measures analysis of variance (rm-ANOVA) method with Bonferroni corrections was used. *P*-values < 0.05 were considered to be statically significant.

## Results

### Cell and Cell Viability

Labeling efficiencies of the macrophage subtypes with (^111^In)In-(oxinate)_3_ were unstimulated cells 51.0 ± 2.9% (3.3 ± 1.3 × 10^6^ cells) (*n* = 3), the LPS/IFNγ activated cells 57.7 ± 0.6% (1.5 ± 0.14 × 10^6^ cells) (*n* = 3), and the IL-4/IL-10/TGFβ activated cells 71.1 ± 0.5% (2.4 ± 0.12 × 10^6^ cells) (*n* = 3), respectively. Yielding a final radioactive cell dosage to unstimulated cells of 6.3 ± 0.7, LPS/IFNγ activated cells of 5.0 ± 0.4, and IL4, IL10/TGFβ1 activated cells received a dose of 2.9 ± 0.6 Bq/cell, respectively. The viability was initially high for unstimulated cells (94.0 ± 0.8%) and IL4, IL10/TGFβ1 activated cells (96.3 ± 0.5%), while LPS/IFNγ activated started with a slightly lower viability (85.7 ± 5.4). The complete procedure of radiolabeling and catheter passage resulted in a decrease in viability for all cell lines. Unactivated cell viability decreased by 5.0 ± 3.1%, LPS/IFNγ activated cells by 10.0 ± 8.2%, and IL-4/IL-10/TGFγ activated cells by 2.1 ± 0.8%. However, there was no significant difference in viability before and after radiolabeling and catheter passage for any cell types (Table 2).

**Table 2.** Viability of Cells Measured During the Procedure of (^111^In)In-(oxinate)_3_ Radiolabeling and Catheter Passage Using Trypan Blue Staining Providing the Percentage of Live Cells.

Cell viability during radiolabeling and catheter passage			
	Unstimulated cells	LPS/IFNγ activated cells	IL4, IL10/TGFβ1 activated cells
Control pre-catheter	94.0 ± 0.8%	85.7 ± 5.4%	96.3 ± 0.5%
Control post-catheter	94.7 ± 1.9%	81.7 ± 2.5%	95.7 ± 2.6%
(^111^In)In-(oxinate)3 pre-catheter	91.7 ± 3.4%	76.7 ± 5.9%	95.7 ± 1.2%
(^111^In)In-(oxinate)3 post-catheter	89.3 ± 3.4%	76.7 ± 2.6%	94.3 ± 0.5%
P-value	0.17	0.17	0.20
Samples size	*n* = 3	*n* = 3	*n* = 3

Measurements were obtained on unlabeled controls before and after catheter passage, as well as radiolabeled cells before and after catheter passage. Data are presented as mean value (%) ± SD, statistical analysis calculated by repeated measures analysis of variance (rm-ANOVA), and statistical significance set to a *p*-value of 0.05. LPS/IFNγ: lipopolysaccharide/gamma interferon; IL4: interleukin 4; IL10: interleukin 10; TGFβ1: transforming growth factor beta 1.

### Phagocytosis

**Figure 2.**
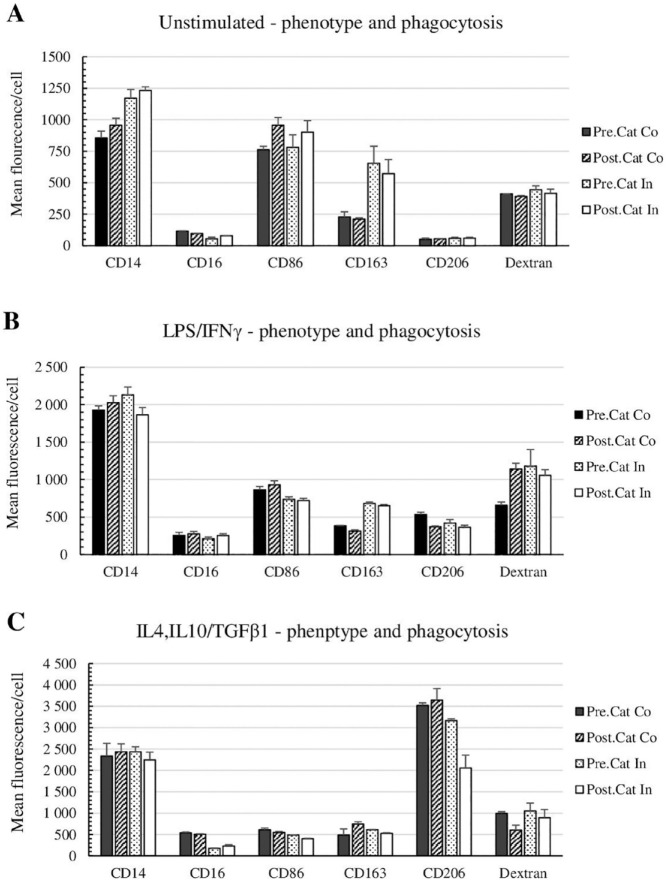
Phenotype analysis and phagocytosis function of (A) M0, (B) LPS/IFNγ activated cells, and **(C)** IL4, IL10/TGFβ1 activated cells measured during each step of the radiolabeling procedure with (^111^In)In-(oxinate)_3_ and catheter passage. An antibody panel of CD14, CD16, CD86, CD163, and CD206 for phenotype and Dextran Alexa 640 for phagocytosis was analyzed by flow cytometry. Statistical analysis was calculated with the Student *t*-test or rm-ANOVA. Data were considered statistically significant at a *p*-value <0.05, values under 0.05 are marked as *, and those under 0.001 are marked as <0.001**. *P*-values are presented in Table 3.

The procedure of radiolabeling and catheter passage had no significant effect on phagocytosis of unstimulated and IL4, IL10/TGFβ1 activated cells, but a weakly significant increased phagocytosis of 62 ± 20% (*p* = 0.049) was recorded in LPS/IFNγ activated cells. Catheter passage significantly affected the surface antigen expression for all cell types. Unstimulated and IL4, IL10/TGFβ1 activated cells decreased by 4.7 ± 1.6% (*p* = 0.06) and 37 ± 27% (*p* = 0.03), respectively. For the LPS/IFNγ activated cells, the phagocytosis increased significantly with 74 ± 10% (*p* = 0.007) (Table 3 and Fig. 2).

**Table 3.** The Percental Change in Antigen Expression and *p*-Values for Phenotype Analysis and Phagocytosis Function of Unstimulated Monocytes, LPS/IFNγ, or IL4/IL10/TGFβ1 Activated Cells Measured During Each Step in the Procedure of Radiolabeling With (^111^In)In-(Oxinate)_3_ and Catheter Passage.

Change in antigen expression and phagocytosis (%) *P*-values for antigen expression and phagocytosis						
Unstimulated monocytes	CD14	CD16	CD86	CD163	CD206	Dextran
Catheter passage *P*-value	+12 ± 6.1% 0.095	–17 ± 2.4% 0.13	+25 ± 3.6% 0.012*	–5 ± 18% 0.65	+12 ± 28% 0.73	–4.7 ± 1.6% 0.011*
Radiolabeling *P*-value	+38 ± 16% 0.065	–52 ± 12% 0.030*	+2.1 ± 9.7% 0.75	+185 ± 25% 0.026*	+26 ± 45% 0.54	+8.1 ± 7.6% 0.13
Complete procedure *P*-value	+45 ± 12% 0.007*	–32 ± 4.1% 0.004*	+18 ± 8.0% 0.05	+150 ± 26% 0.003*	+24 ± 32% 0.53	+1.3 ± 7.8% 0.18
LPS/IFNγ activated cells	CD14	CD16	CD86	CD163	CD206	Dextran
Catheter passage *P*-value	+5.2 ± 5.7% 0.33	+9.6 ± 7.2% 0.18	+8.1 ± 9.9% 0.37	–18 ± 4.9% 0.037*	–30 ± 2.5% 0.009*	+74 ± 10% 0.007*
Radiolabeling *P*-value	+11 ± 6.4% 0.14	–17 ± 11% 0.19	–15 ± 2.9% 0.024*	+77 ± 4.5% 0.002*	–21 ± 11% 0.12	+78 ± 22% 0.054
Complete procedure *P*-value	–3.2 ± 4.8% 0.39	+0.14 ± 8.0% 0.94	–17 ± 3.8% 0.003*	+70 ± 5.9% <0.001**	–32 ± 8.8% 0.041*	+62 ± 20% 0.049*
IL4, IL10/TGFβ1 activated cells	CD14	CD16	CD86	CD163	CD206	Dextran
Catheter passage *P*-value	+6.4 ± 19% 0.78	–7.6 ± 0.4% 0.11	–10 ± 9.6% 0.30	+71 ± 59% 0.11	+3.5 ± 7.3% 0.57	–37 ± 27% 0.030*
Radiolabeling *P*-value	+5.5 ± 10% 0.59	–68 ± 1.0% 0.002*	–20 ± 5.5% 0.049*	+41 ± 50% 0.33	–10 ± 1.9% 0.021*	+5.8 ± 20% 0.073
Complete procedure *P*-value	–1.9 ± 17% 0.87	–57 ± 6.5% <0.001**	–34 ± 3.7% 0.002*	+22 ±45% 0.39	–42 ± 7.8% 0.61	–10 ± 22% 0.98

Each procedure is divided into three analysis groups; “Catheter passage” unlabeled cells before and after catheter passage; “Radiolabeling” unlabeled cells compared with radiolabeled cells; “Complete procedure” unlabeled cells versus radiolabeled cells after catheter passage, to evaluate the whole procedure of radiolabeling and cell delivery *in vivo.* An antibody panel of CD14, CD16, CD86, CD163, and CD206 for phenotype and Dextran for phagocytosis was analyzed by flow cytometry. Statistical analysis was calculated with the Student *t*-test or rm-ANOVA. Data were considered statistically significant at a *p*-value <0.05, values under 0.05 are marked as *, and those under 0.001 are marked as <0.001**.

LPS/IFNγ: lipopolysaccharide/gamma interferon; IL4: interleukin 4; IL10: interleukin 10; TGFβ1: transforming growth factor beta 1.

### Phenotyping

Monocytes naturally have a lower antigen expression and are easily differentiated through activation. The complete procedure of radiolabeling and catheter passage resulted in a substantial increase in antigen expression on unstimulated monocytes, including CD14 of 45 ± 12% (*p* = 0.007) and CD163 of 150 ± 26% (*p* = 0.003), and a slight increase of CD86 with 18 ± 8.0% (*p* = 0.05), while CD16 decreased significantly with 32 ± 4.1% (*p* = 0.004) (Table 3 and Fig. 2). However, the main reason for the change in antigen expression was caused by radiolabeling. Catheter passage only significantly affected the CD86 expression with a 25 ± 3.6% decrease (*p* = 0.012).

This procedure had no significant effect on LPS/IFNγ activated cell expression of CD14 and CD16 but caused a significant decrease of CD86 levels with 17 ± 3.8% (*p* = 0.003) and of CD206 expression with 32 ± 8.8% (*p* = 0.04) for which the catheter passage had a large impact. Only CD163 levels were substantially increased by 70±5.9% (*p* < 0.0001), solely caused by radiolabeling.

**Figure 3.**
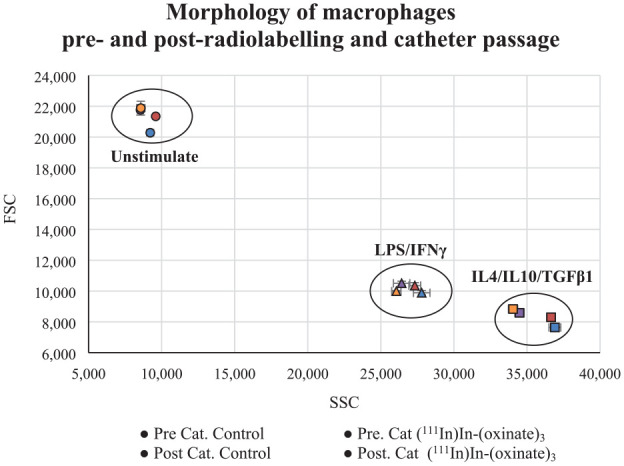
Flow cytometry parameters sideway scatter (SSC) and forward scatter (FSC). SSC represents the intracellular structures providing information on the internal complexity of the cells. The FSC represents the cell size. Combined they provide the basic information of the cell morphology of unstimulated, LPS/IFNγ, or IL4/IL10/TGFβ1 activated cells. Values are presented as the mean SSC/FSC value for each step in the process; pre-catheter or post-catheter passage for controls and (^111^In)In-(oxinate)_3_ labeled cells.

The overall procedure had a small impact on the phenotype expression for IL4, IL10/TGFβ1 activated cells. There were no significant changes in the expression of CD14, CD163, and CD206 (Table 3 and Fig. 2). There was, however, a significant loss in the expression of CD16 (57 ± 6.5%, *p* ≤ 0.0001) and CD86 (34 ± 3.7%, *p* = 0.002) primarily caused by radiolabeling. Although IL4, IL10/TGFβ1 activated cells have a naturally lower expression of both CD16 and CD86, it could be considered within the expression range of the phenotype. All cell types stayed within their respective range of FSC (density) and SSC (size) (Fig. 3).

### Cell Transplantation

**Figure 4.**
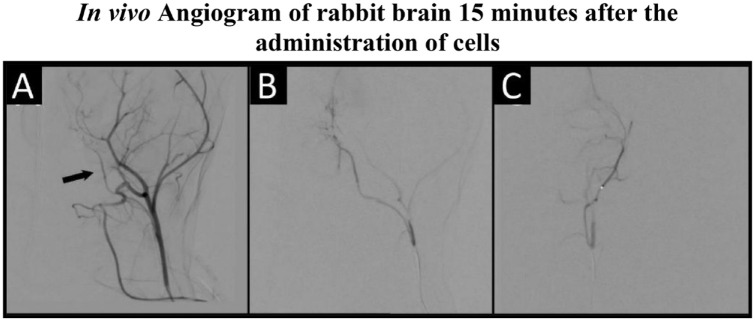
A representative digital subtraction angiogram taken 15 min after administration of LPS/IFNγ activated cells. Images are taken in the following order: the right internal carotid artery (ICA) (arrow, A) followed by selective ICA arteriograms in lateral (B) and frontal (C) planes.

**Figure 5.**
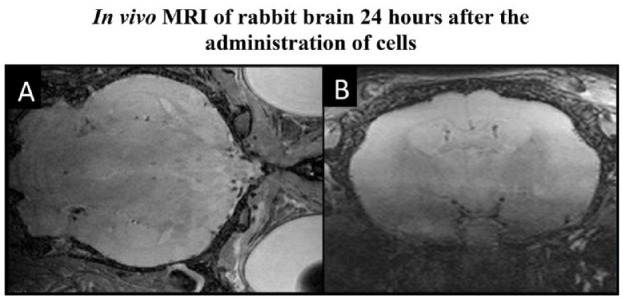
Volumetric MRI images, (A) T2- and (B) Diffusion-weighted, were taken 24 h post-cell administration of LPS/IFNγ activated cells through the catheter to rabbit brain. The illustration shows no adverse events caused by the procedure.

Surgical anesthesia and intubation were successfully achieved in all animals. Endovascular access was established in all animals. No acute adverse events occurred during endovascular navigation to the internal carotid artery. As verified by digital subtraction angiography, no thromboembolic events occurred during or after IA cell administration (Fig. 4). Animals were continuously monitored during infusion using a gamma counter. Fifteen minutes after cell administration, no radiotracer signal could be detected in the brain. Additionally, after 24 h, MRI further confirmed the absence of acute infarcts evaluated by DWI (*n* = 3 for LPS/IFNγ activated cells and *n* = 3 for IL4, IL10/TGFβ1 activated cells) (Fig. 5). The six animals receiving non-radioactive cells were then sacrificed after the completion of imaging on day 0.

**Figure 6.**
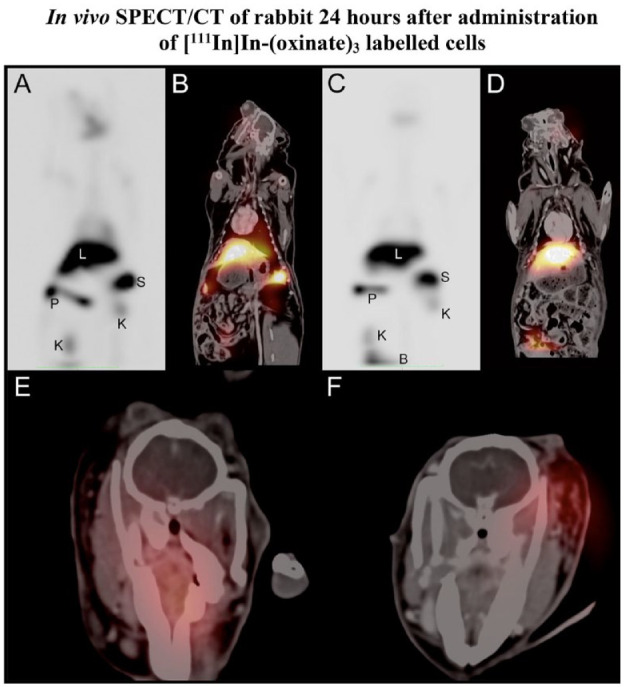
SPECT coronal maximum intensity projections for the full rabbit scan 24 h following administration of LPS/IFNγ activated cells (A) and IL4, IL10/TGFβ1 activated cells (C). Liver indicated by L, spleen by S, pancreas by P, kidneys by K and in panel C, and the top of the bladder marked by B. Coronal SPECT-CT fusion images demonstrating the colocalization of signal for LPS/IFNγ activated cells (B) and IL4, IL10/TGFβ1 activated cells (D) to organs. Axial SPECT-CT fusion images demonstrate the lack of signal from the brain parenchyma for both LPS/IFNγ activated cells (E) and IL4, IL10/TGFβ1 activated cells (F), and the visible signals are due to accumulations in glands.

An additional two animals received either LPS/IFNγ activated cells (*n* = 1) or IL4, IL10/TGFβ1 activated cells (*n* = 1). The same procedure of angiography and MRI was conducted at the time of injection. These animals were then extubated and allowed to return to their cages. After 24 h, animals were sedated, intubated, and imaged by MRI and SPECT/CT. The MRI was performed using a high-resolution T2-weighted volumetric scan (voxel size 200 × 267 × 267 µm) in addition to DWI. The gamma counter showed a quick clearance of signal from the brain after cell administration. After 24 h, no signal could be detected in the brain with SPECT/CT imaging, indicating a complete passage through the brain without retention of administered cells (Fig. 6). The majority of the SPECT signal was detected in the liver, the spleen, and to some extent, the kidneys and bladder. No signal was detected in the lungs.

## Discussion

Cell-based therapy administered by IV injection is the golden standard for many different types of treatment, such as cancer and autoimmune diseases^5–11^. Nonetheless, several treatments suffer from side effects and unpredictable treatment efficiency due to off-target accumulation of cells when using IV or IA. Other types of injection for a more direct tissue delivery have been evaluated for several diseases. Cell delivery by SC has been used to treat Diabetes type 1, intramuscular (IM) to treat amyotrophic lateral sclerosis (ALS), and post-organ transplantation treatment with intraosseous (IO) injections to mention a few. Although this avoids the passage through the lungs and liver, other limitations emerge. It has been shown to increase the risk of an aggressive immune response in the sight of injection as well and the cells’ capacity for tissue penetration and mobilization is limited compared with IV and IA injections. The delivery of cells into enclosed tissues shows a high risk of cell clogging and a high level of cell death^43–46^. By using alternative administration methods with targeted cell delivery via an IA catheter, it is possible to avoid accumulation in the lungs and liver, hence increasing the proportion of cells reaching the target tissue. However, this delivery system needs to be evaluated to ensure that the procedure itself does not damage the cells or cause severe damage to brain tissue due to the invasive penetration of the catheter. In this study, we evaluated the effects on phenotypic expression and phagocytic function of unstimulated monocytes and activated macrophages after radiolabeling with (^111^In)In-(oxinate)_3_ followed by catheter passage *in vitro*. Subsequently, we evaluated the safety by administering cells *in vivo* to rabbit brains, the animals being assessed using SPECT and MRI imaging to locate the cells and to identify potential complications from the catheter navigation.

The procedure of radiolabeling and catheter passage entailed no significant difference in cell viability for any of the cell types. One of the batches of LPS/IFNγ activated cells showed a slightly lower viability compared with the other cell types, hence the higher SD. This is, however, not unlikely when handling immune cells due to their sensitivity, therefore, there was no need to exclude them from the study. The radioactive cellular retention of [^111^In]In-(oxinate)_3_ has previously been reported and has shown that [^111^In]In-(oxinate)_3_ can be unpredictable. In general, [^111^In]In-(oxinate)_3_ labeled cells retain 60–85% of the radioactivity after 24–48 h^34–36^. What all studies disclose is that retention is determined by the health of the cells, hence with high cell viability the radioactive efflux is minimized.

Monocytes can differentiate into the desired activated macrophage type when exposed to different stimuli; LPS/IFNγ which induces a primarily pro-inflammatory activation phenotype or IL4/IL10/TGFβ1 which induces a primarily immunomodulatory activation phenotype (Table 1).

The antigen expression panel for surface phenotyping was designed to distinguish the subtype-specific antigen expression (Table 1). The cell line-specific antigen CD14 is expressed on all macrophage subtypes, and its primary function is to detect and bind foreign antigens (bacteria, viruses, and parasites)^37^. CD16 has an overall low expression on monocytes and macrophages, separating them from other CD16^+^ cells such as large granular lymphocytes, T-cells, and NK cells^38^. CD86 is a T-cell activation antigen highly expressed on LPS/IFNγ activated cells and moderately expressed on unstimulated cells. The IL4/IL10/TGFβ1 activated cells have a low expression of CD86 and CD163^39–41^. The inflammation type 2 associated antigen CD206 is activated explicitly by parasites and is therefore solely expressed on IL4/IL10/TGFβ1 cells^42^. The unstimulated cells generally have lower surface antigen expression and hence reduced phagocytic abilities.

The unstimulated cells were strongly affected by radiolabeling and catheter passage, with a significant change in all surface antigen expressions except for CD206. The shift in CD163 levels is a strong indicator of inflammatory activation^40,41^. The lack of increase in CD206 also supports that the unstimulated cells differentiated toward a pro-inflammatory LPS/IFNγ activated phenotype^42^. The high increase in CD14, although usually expressed on unstimulated cells, also points toward differentiation to activated macrophage phenotypes^37^. Unstimulated cells that passed through the catheter showed a significant decrease in phagocytic function, yet radiolabeled cells showed an increasing effect; however, the completed procedure had no significant effect on phagocytosis. The catheter passage had no significant effect on IL4/IL10/TGFβ1 cells, while the radiolabeling procedure had apparent adverse effects on these cells. The morphological appearance of unstimulated and IL4/IL10/TGFβ1 activated cells have an elongated shape, hence a larger variety in SSC and FSC, while LPS/IFNγ activated cells have a rounded shape, hence lower deviations^47^.

The standard procedure to administer transplanted cells is IV infusions and has proven very successful in numerous treatments due to the quick biodistribution through the bloodstream and extensive tissue penetration. However, this strength is also a weakness. A particular challenge during cell migration via this route is the passage through the lungs, followed by the high blood flow through the liver. Indeed, several preclinical studies of nuclear cell tracking *in vivo* following IV injection have demonstrated that some cells linger in the lung and have high accumulation in the liver and spleen. Which subsequentially increased organ dosage, a lower target-to-background ratio and fewer cells available to migrate to the intended tissue^18,33,48–53^. A portion of the liver and spleen signal is caused by dead cells and radiotracer leakage, as discussed above. Furthermore, not only does the off-target accumulation reduce the treatment efficacy, administration of stem cells and immune cells such as CAR T-cells has been shown to cause severe side effects such as graft-versus-host disease^4,54–56^. Hence, developing and evaluating the alternative delivery system of IA catheter delivery would substantially contribute to cell-based therapy, especially when the target tissue is difficult to access, like the brain, or is in proximity to the lungs or liver.

We further performed *in vivo* experiments to establish safety data for the interaction between intra-arterially administered activated cells and the healthy brain parenchyma. As no cell retention could be visualized in the brain by SPECT/CT after 24 h, the epithelium of the healthy brain does not attract the activated cells. The signal was mainly detected in the liver and spleen which was expected since the cells migrated threw the artery and first reached the liver instead of the lungs. Immediate side effects such as thromboembolism were excluded by the normal appearance of the arterial tree on DSA following cell administration and confirmed by lack of acute infarction on MRI after 24 h. In addition, there were no delayed side effects such as cerebral infarcts or edema, on high-resolution 3D T2-weighted images at 24 h when using the cell transplantation parameters in this study. This is essential baseline information for further clinical translation of macrophage cell transplantation to the injured central nervous system.

## Conclusion

IA selective macrophage injection by microcatheter technique to the brain vasculature does not cause ischemic or other adverse events and the intact blood–brain barrier of the normal brain prevents cell diapedesis. Radiolabeling of cells *in vitro* and administration by a catheter *in vivo* caused different effects on cell activation phenotypes. Both radiolabeling and catheter passage showed significant changes in all cell types. Despite this change, both LPS/IFNγ activated cells and IL4/IL10/TGFβ1 macrophages stay within their natural phenotypic pattern range. We propose that they are thus suitable candidates for targeted administration via IA transplantation. The unstimulated monocytes showed signs of activation, especially toward LPS/IFNγ activated cells. As unstimulated monocytes are highly sensitive and easily activated and, from this study, are not an appropriate choice for radiolabeling and cell transplantation using this administration technique. The consequences of an increased inflammatory response at the target site should be considered when administering immune cells. Monocytes that undesirably differentiate into LPS/IFNγ activated cells can cause tissue damage, while IL4/IL10/TGFβ1 cells will trigger tissue repair. These effects can both be desirable or cause severe consequences depending on the therapeutic application.
